# A Novel Arsenate-Resistant Determinant Associated with ICEpMERPH, a Member of the SXT/R391 Group of Mobile Genetic Elements

**DOI:** 10.3390/genes10121048

**Published:** 2019-12-16

**Authors:** Michael P. Ryan, Shannon Slattery, J. Tony Pembroke

**Affiliations:** Department of Chemical Sciences and Bernal Institute, School of Natural Sciences, University of Limerick, V94 T9PX Limerick, Ireland; Shannon.Slattery@ul.ie (S.S.); Tony.Pembroke@ul.ie (J.T.P.)

**Keywords:** mobile genetic elements, integrative conjugative elements (ICEs), R391, pMERPH, SXT

## Abstract

ICEpMERPH, the first integrative conjugative element (ICE) of the SXT/R391 family isolated in the United Kingdom and Europe, was analyzed to determine the nature of its adaptive functions, its genetic structure, and its homology to related elements normally found in pathogenic *Vibrio* or *Proteus* species. Whole genome sequencing of *Escherichia coli (E. coli)* isolate K802 (which contains the ICEpMERPH) was carried out using Illumina sequencing technology. ICEpMERPH has a size of 110 Kb and 112 putative open reading frames (ORFs). The “hotspot regions” of the element were found to contain putative restriction digestion systems, insertion sequences, and heavy metal resistance genes that encoded resistance to mercury, as previously reported, but also surprisingly to arsenate. A novel arsenate resistance system was identified in hotspot 4 of the element, unrelated to other SXT/R391 elements. This arsenate resistance system was potentially linked to two genes: *orf69*, encoding an organoarsenical efflux major facilitator superfamily (MFS) transporter-like protein related to ArsJ, and *orf70*, encoding nicotinamide adenine dinucleotide (NAD)-dependent glyceraldehyde-3-phosphate dehydrogenase. Phenotypic analysis using isogenic strains of *Escherichia coli* strain AB1157 with and without the ICEpMERPH revealed resistance to low levels of arsenate in the range of 1–5 mM. This novel, low-level resistance may have an important adaptive function in polluted environments, which often contain low levels of arsenate contamination. A bioinformatic analysis on the novel determinant and the phylogeny of ICEpMERPH was presented.

## 1. Introduction

Integrative conjugative elements (ICEs) are a family of bacterial mobile genetic elements characterized by their ability to facilitate their own integration, excision, and transfer from one bacterial host genome to another. They achieve this via site-specific recombination, self-circularization, and conjugative transfer [1]. One of the largest and most-studied families of ICEs is the SXT/R391 family (named after the first two of these elements to be sequenced). This family of elements has >60 members, the elements being identified either experimentally or bioinformatically to date [2]. There are also various subtypes of SXT/R391 elements [3]. Type 1 elements have been found in a variety of Gammaproteobacteria species, including the *Vibrio*, *Shewanella, Proteus*, and *Photobacterium* (a full list can be found in Supplementary Table S1 [3,4,5,6,7,8,9,10,11,12,13,14,15,16,17,18,19,20,21,22,23,24,25,26,27,28,29,30,31,32,33,34,35,36,37,38,39,40,41,42,43,44,45,46,47,48,49,50,51,52,53]). They are distinguished by possessing a type 1 integrase, which allows for site-specific integration into the 5′ end of the essential *prfC* gene [54]. Integration restores a functioning *prf*C gene and encodes a new hybrid PrfC protein [54]. Type 2, 3, and 4 ICEs all insert at the 3′ end of the multicopy tRNA-Ser gene and have been found solely in *Vibrio* species [3,55]. Another type of SXT/R391 ICE integrates into the *pabA* (para-aminobenzoate synthase) gene as ICE*Sh*95 [38]. ICER391 is the first element of this family, discovered in a *Providencia rettgeri* clinical isolate from South Africa in 1967 [56], and encodes genes for resistance to kanamycin and mercury [3,4].

Structurally, the SXT/R391 family contains 51 near-identical core genes, many of which are involved in integration/excision, conjugative transfer, and regulation of the ICEs [26,57,58,59,60]. In addition to these core genes, all elements contain five hotspots (called HS1–5) and up to five variable regions (called VRI-V) where accessory genes, such as antibiotic resistance genes, heavy metal resistance genes, or DNA repair genes, can be found inserted [6,10].

SXT/R391 ICEs allow bacteria to adapt and survive in a variety of environmental niches by encoding various heavy metal resistance genes, antimicrobial resistance genes, and bacteriophage defense genes. ICEs of the SXT/R391 family have been shown to encode resistances to various heavy metals, including cobalt (Co), cadmium (Cd), mercury (Hg), and zinc (Zn), which in some cases are related to multi-efflux systems, as identified in ICE*Spu*PO1 [14]. This ICE encodes an efflux pump (a member of the integral membrane protein family) found to give greater tolerance levels to divalent metal ions, including cadmium, zinc, and cobalt, by removing the metal ions from the cells [61,62]. This ICE has also been found to contain a transcriptional regulator for arsenic (but no resistance determinant) and a mercury resistance operon located in variable region IV from ORF 83–88 [10]. Mercury resistance operons have been found in many SXT/R391 ICE elements, the most notable being ICER391 [4] and ICEpMERPH (the subject of this study) isolated from *Shewanella putrefaciens* [63]. Mercury operons have also been discovered in ICE*Vsp*Spa1 and ICE*Eni*Spa1 from marine aquaculture in Spain and ICE*Vsp*Por1 and ICE*Vsp*Por3 from Portugal [64], while several *Proteus mirabilis* isolates from China have also been found to contain mercury resistance determinants [12]. ICE*Sh*95 from a *Shewanella* species and ICE*PrSt*33672 from *Providencia stuartii* ATCC33772, both clinical isolates, have also been shown to encode a mercury operon [28,65]. Complete arsenic operons have not been previously identified in SXT/R391 ICE elements but have been detected in several other types of unrelated ICEs. ICE*Sde*3396, from *Streptococcus dysgalactlae*, contains a cadmium resistance and an arsenic resistance operon [66], and several Tn*4371*-like ICEs also have been found to contain arsenic resistance operons [67].

In this study, the nucleotide sequence of the SXT/R391 ICEpMERPH, originally isolated from the River Mersey in 1987 [63], which had been left highly polluted as a result of the industrial revolution [68], is determined and analyzed. It has previously been shown to encode a mercury resistance operon similar [63] to that encoded by ICER391 [4], but as limited analysis has been subsequently carried out on the element, it has been believed that this is the only resistance encoded. 

## 2. Materials and Methods 

### 2.1. Bacterial Strains

The bacterial strains and mobile genetic elements used in this study are listed in Table 1. Strains were stored at −80 °C in Luria-Bertani (LB) broth containing 50% glycerol. For most experiments, *E. coli* cultures were grown aerobically in LB broth.

### 2.2. Genome Sequencing and Annotation

The genome of *Escherichia coli* isolate NCIMB12504 (K802) (which contains ICEpMERPH) was sequenced by MicrobesNG (University of Birmingham, Birmingham, UK) using paired-end (insert size between the ends 200–500 bp) HiSeq2000 Illumina technology giving approximately 30-fold coverage. The resulting reads were processed and assembled into 72 contigs using MicrobesNG’s own automated analysis pipeline. The ICEpMERPH genome was identified amongst 72 contigs by using the basic local alignment search tool (BLAST) to investigate the presence of several different ICER391 (AY090559), ICER997 (KY433363), and ICESXT (AY055428) core scaffold genes (*int, xis, traLEKBVA*, and *setCD*). The element was found on a single contig. The ICEpMERPH sequence was then annotated using the RAST (Rapid Annotation using Subsystem Technology) server and the BLAST program at the National Center for Biotechnology Information (NCBI) [69,70]. Putative functions for all proteins were inferred using BLAST (http://ncbi.nlm.nih.gov/BLAST) or InterPro Scan (https://www.ebi.ac.uk/interpro/).

### 2.3. Phylogenetic Analysis of Core ICE Genes 

Phylogenetic analysis of ICEpMERPH was performed based on comparison with the concatenated amino acid sequences of 48 SXT/R391 core ICE gene-encoded proteins on all previously sequenced whole SXT/R391 elements. These elements were listed in Supplementary Table S3. An unrooted phylogenetic tree was constructed by the maximum-likelihood method based on the Poisson correction model using MEGAX [71]. Bootstrap analysis with 1000 replications was performed to test the reliability of the tree.

### 2.4. Accession Number

ICEpMERPH was submitted to GenBank under accession number MH974755.

### 2.5. Phenotypic Testing

ICEpMERPH was transferred to *E. coli* strain AB1157 (from *E. coli* K802) via the method outlined in Murphy and Pembroke, 1995 [72]. Both AB1157 and AB1157ICEpMERPH were then tested for their susceptibility to two arsenic-based compounds: sodium arsenate dibasic heptahydrate (Na_2_HAsO_4_.7H_2_O) and sodium meta-arsenite (NaAsO_2_). A stock solution of 1 M arsenate and 100 mM arsenite was prepared. A range of different dilutions were prepared: 1 mM, 5 mM, 10 mM, and 20 mM, using a modified low-phosphate media (80 mM NaCl, 20 mM KCl, 20 mM NH_4_Cl, 3 mM NH_4_SO_4_, 1 mM MgCl_2_, 2 mM ZnCl_2_, and 0.12 M Tris base, supplemented with 0.5% (w/v) glucose, 2 mg/mL thiamine, 1% peptone, and 0.1 mM CaCl_2_ adjusted to pH 7.0–7.4 with hydrochloric acid (HCL) [73]). All testing was carried out using low-phosphate media, as the higher levels of phosphate in media (such as LB) can reduce the toxicity of arsenate [73]. Additionally, 1% of casamino acids were added to the media as a modification in order to help growth. The samples were tested in 96 well microtiter plates, with blanks included. The microtiter plates were incubated at 37 °C with a slight shake for a period of 60 h on a Biotek ELx808 Ultra microplate reader (Mason Technologies, Dublin, Ireland). Readings were taken every hour at 595 nm.

## 3. Results and Discussion

### 3.1. Full Sequence Analysis

**Figure 1:**
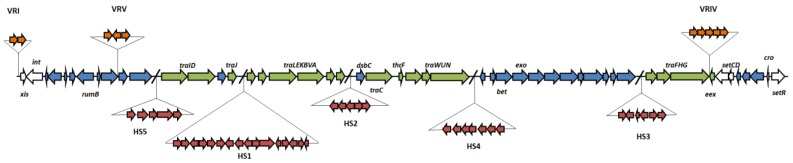
Molecular map of ICEpMERPH displaying the location of genes associated with the 110 Kb mobile genetic element. Genes colored white are associated with excision, integration, and control. Genes colored green are associated with transfer. All other core genes are colored blue. Genes associated with hotspots are colored red, and those associated with variable regions are colored orange.

ICEpMERPH can be classified as a type 1 SXT/R391 ICE based on comparative genomics of the newly determined nucleotide sequence. Analysis of the annotated sequence revealed the ICEpMERPH has 112 open reading frames (ORFs) and follows the conserved synteny for “typical” type 1 R391/SXT elements (Figure 1). A full gene list can be seen in Supplementary Table S3, with comparisons to ICER391 and ICESXT. Fifty-one of these ORFs were predicted to code for the core scaffold of SXT/R391 elements (genes related to integration, excision, conjugative transfer, and regulation) [3]. All other genes were found in the hotspots and variable regions of the ICEpMERPH genome (Figure 1).

ICEpMERPH hotspot 1 (HS1) contains the same 18-gene insertion as previously found in HS1 in ICE*Mpr*Chn1 (*orf32* to *orf47*), showing 94% to 100% nucleotide identity across all genes [8]. Most of these genes encode hypothetical proteins of unknown functions that have been conserved only in these type of elements. There are several predicted transposases and genes putatively encoding a three-component efflux pump, based on their low homology level (CDD Blast) with the *acr*AB efflux system [74]. In order to determine if any antimicrobial resistance could be related to this efflux pump, a panel of antibiotics (Supplementary Table S2) and the antibacterial triclosan were tested against AB1157ICEpMERPH. No increased level of resistance was detected to triclosan or with the drug panel used (Supplementary Table S2).

ICEpMERPH hotspot 2 (HS2) contains five genes also of unknown function. The first three genes are highly similar to those found in HS2 of ICE*Vch*Mex01 [10]. Four of the five genes share similarity to those found in HS2 of ICE*Vpa*Can1 [53]. 

The insertion in hotspot 3 [HS3] is made up of six predicted ORFs. The hotspot encodes an interrupted *mcr*BC-like restriction digestion system. This system was originally discovered in *E. coli* K-12 [75]. The *mcrB* gene is interrupted by the insertion sequence IS*Pst2*b. IS*Pst2*b is made up of three genes. The first encodes an ISL-3 transposase, the second an ArsR-like transcriptional regulator, and the third a permease [76]. The functions of the non-transposase genes in this insertion sequence are unknown. This structure is found also in Tn*6516*, originating from *Achromobacter* spp, and in ICE*H*s1 (a non-SXT/R391 ICE), an element found in *Histophilus somni* that encodes for antimicrobial resistance and metal tolerance [77]. Following this insertion is the rest of the truncated *mcrB* gene and the entire *mcrC* gene. ICE*Pmi*Jpn1 has been shown to contain a similar uninterrupted *mcrBC* restriction digestion system [32,78]. 

The insertion in hotspot 5 [HS5] codes for the putative type I restriction-modification system (RM) *hds*RMS [79,80] similar to that found in ICE*Vch*Mex01 [10]. These systems carry out DNA modification, recombination, and repair and are composed of three polypeptides: R (restriction endonuclease), which recognizes and cuts specific DNA sequences; M (modification), which methylates the same sequence to inhibit DNA cleavage and protect the host cell against invasion of foreign DNA; and S (specificity), which determines the specificity of both R and M [80]. These genes may confer protection against bacteriophage infection, as was demonstrated for other ICEs of the SXT/R391 family [79]. In both cases, there is a gene inserted between *hdsS* and *hdsR* in ICEpMERPH, encoding a nuclease of unknown cellular function.

ICEpMERPH contains no insertions in variable regions II and III (VRII and VRIII). The element does, however, have insertions in variable regions I, IV, and V (VRI, VRIV, and VRV). The insertion in VRI is structurally identical to that found in the ICE R391. VRI contains three genes, including a putative *hip*AB-like toxin-antitoxin (TA) system. This system improves stability of the element when integrated into the bacterial chromosome. A Δ*hipA* mutant of ICER391, shows a 12-fold increase in loss of the ICE from the host when compared to the wild-type [81]. VRIV contains a five-gene mercury resistance system (*merRTPCA*). The *merR* encodes a regulatory sequence; *merA* encodes a detoxifying oxido-reductase; and *merC*, *merT*, and *merP* encode transport proteins. This system is also found in ICER391, ICE*Pmi*Chn2410, and ICE*Pmi*Chn2416 [4,12,82]. VRV contains four genes that share homology with VRV of ICE*Val*A056-2. The potential function of this variable region is unknown [15].

**Figure 2:**
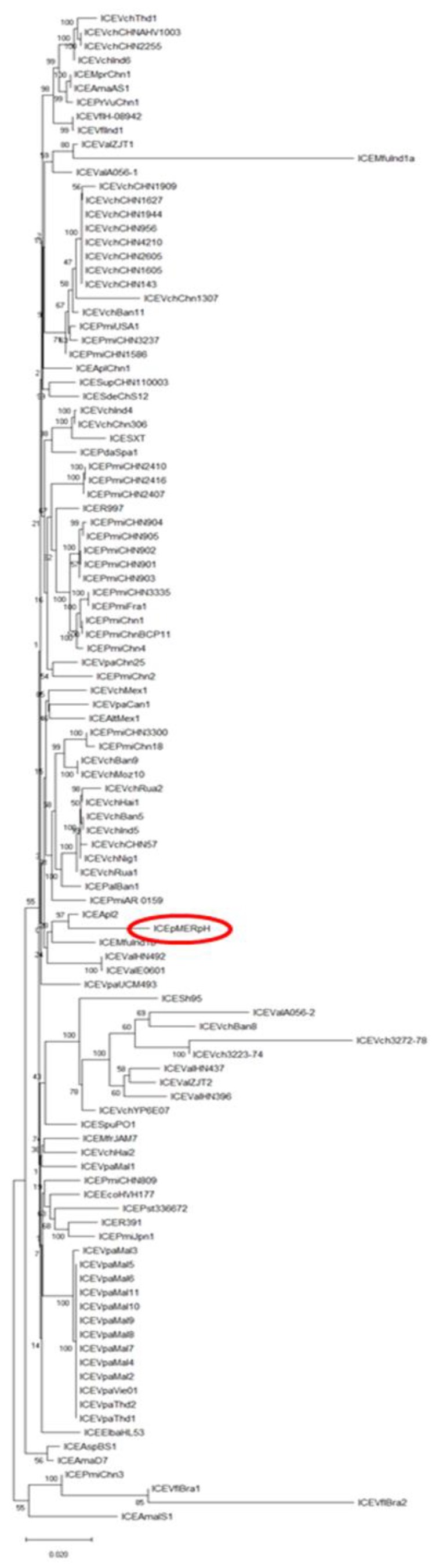
Phylogenetic tree from the maximum-likelihood analysis of the core concatenated proteins of 111 SXT/R391 ICEs.

A phylogenetic tree (Figure 2) was constructed based on the concatenated amino acid sequences of all SXT-R391 core proteins for most published core genome sequences of these elements. These elements were found in bacteria from a wide variety of environmental niches and geographic locations. The ICEpMERPH clustered with ICE*Apl*2, which was an ICE discovered in *Actinobacillus pleuropneumoniaei* MIDG3553 that was isolated from the pneumonic lung of a pig [24]. The hotspots of this element are completely different to those found in ICEpMERPH. These results show the wide geographic spread of SXT/R391-like elements that still all retain a highly homologous core molecular structure).

### 3.2. Genetic Basis for Arsenic Resistance Encoded by pMERPH

The insertion in hotspot 4 (HS4) contains a predicted seven-gene insertion. The first two genes encode for a putative arsenic resistance system that bears similarity to that found in *Pseudomonas aeruginosa* DK2. In *P. aeruginosa* DK2, this pathway contains a two-gene system made up of the *gapdh* (65% similarity to the ICEpMERPH ORF) and *arsJ* (60% similarity) genes. The gene *gapdh* encodes a predicted glycolytic enzyme, glyceraldehyde-3-phosphate dehydrogenase (GAPDH), which is NAD+-dependent. Via this system, inorganic As(V) is transformed into the organoarsenical compound 1-arseno-3-phosphoglycerate (1As3PGA) [83]. This compound is highly unstable. 1As3PGA can be expelled from the cell by the efflux permease a*rsJ*, where it swiftly dissociates into inorganic As(V) and 3-phosphoglycerate (3PGA) due to its short half-life in the natural environment [83].

These genes in ICEpMERPH are followed by a putative tyrosine phosphatase and a thioredoxin-like protein, which are both of unknown cellular function. In some arsenic resistance systems, thioredoxin is frequently used in conjunction with ArsC enzymes, such as the *ars*C of *Staphylococcus aureus* pI258, as an electron source [84]. The next gene is a putative *arsP* that codes for a methylarsenite (MAs[III]) efflux permease that extrudes trivalent organoarsenicals from cells, conferring resistance [85]. The next putative gene codes for an *Acr*3-like protein. *Acr*3 is an arsenite efflux pump, which can pump inorganic arsenite from cells and is found in bacteria, archaea, fungi, and some plant species [86,87]. The final gene in this hotspot codes for a putative *Ars*R family transcriptional regulator. No *arsC-*like genes or similar arsenic reductase genes could be found in this hotspot. Near-identical hotspots have been found in unidentified ICEs in *Vibrio parahaemolyticus* species isolated from diseased seafood in Vietnam and Cambodia, but this is the first time to our knowledge that a system such as this has been identified in an SXT/R391 ICE mobile genetic element. A comparison of this system to other arsenate resistance systems can be seen in Supplementary Figure S1, [66,83,85,88,89,90,91,92,93,94,95,96].

### 3.3. Phenotypic Testing

**Figure 3:**
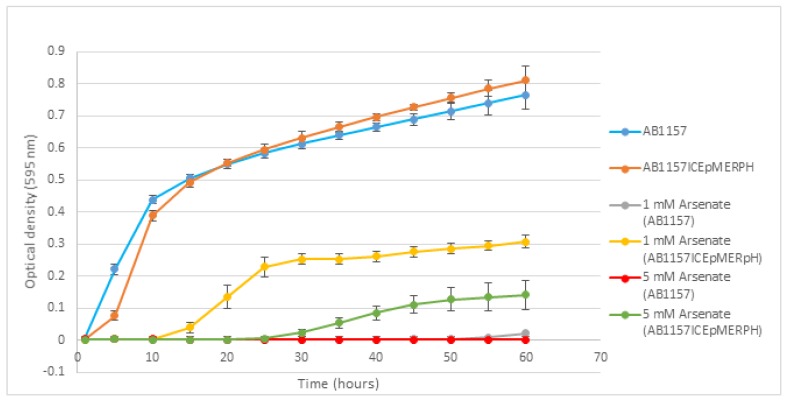
Growth curve of AB1157ICEpMERPH and AB1157 over a 60 h growth period, with arsenate supplemented into the low-phosphorus media.

On identifying a putative arsenic resistance determinant based on the nucleotide sequencing of ICEpMERPH, testing was carried out using AB1157ICEpMERPH and the isogenic strain AB1157 to determine if resistance was phenotypically present. A range of different arsenate concentrations were tested (1–25 mM), and resistance was observed relative to isogenic controls (1–5 mM). Growth analysis over a 60 h period (Figure 3) at 1 mM revealed AB1157 was inhibited, whereas the presence of the ICEpMERPH allowed growth to continue. This was also found to be true at a much lesser extent at 5 mM. No resistance to arsenite was detected at any concentration.

## 4. Conclusions

ICEpMERPH was the first SXT/R391 element identified in the British Isles and has not been previously sequenced. This element contains features found in a variety of SXT/R391 elements from around the globe, indicating the mosaic nature of these elements. The element included a novel arsenate-resistant determinant, the first identified arsenate resistance determinant found associated with SXT/R391 ICE mobile genetic elements. This novel system may give the ICE host an adaptive advantage that allows for survival in polluted environments and allows the maintenance and spread of the element throughout the environment.

## Figures and Tables

**Table 1:** *Escherichia coli (E. coli)* strains used in this study. NCIMB = The National Collection of Industrial, Food, and Marine Bacteria.

Strain	Genotype	Source
AB1157	F^−^, *thr-1*, *araC14*, *leuB6*,∆(*gpt-proA*)62, *lacY1*, *tsx-33*, *qsr’-0*, *glnV44*, *galK2*, *λ-*, *Rac-0*,*hisG4*, *rfbC1*, *mgl-51*, *rpoS396*, *rpsL31* (StrR), *kdgK51*, *xylA5*, *mtl-1*, *argE3*, *thi-1*	*E. coli* genetic stock center (CGSC), Yale University, New Haven, Connecticut, USA
NCIMB12504 (K802)	*F-*, *lacY1 or Δ(cod-lacI)6*, *glnX44(AS), galK2(Oc)*, *galT22*, *λ-*, *e14-*, *mcrA0*, *rfbC1*, *metB1*, *mcrB1*, *hsdR2*	NCIMB

## Supplementary Materials

The following are available online at https://www.mdpi.com/2073-4425/10/12/1048/s1. Table S1. Identified ICESXT/R391 family members with complete genome sequence available incorporated into Figure 3. Table S2: Disk diffusion test results determined by reference to the EUCAST clinical breakpoint tables for enterobacterales. Table S3: Genes of ICEpMERPH. Figure S1: Common arsenic-resistant systems.
