# Supplementary material for: A Novel Arsenate-Resistant Determinant Associated with ICEpMERPH, a Member of the SXT/R391 Group of Mobile Genetic Elements
Source: Genes (Basel). 2019 Dec 16;10(12):1048. doi: 10.3390/genes10121048 (PMC6947025; doi:10.3390/genes10121048)
Supplement: Supplementary file 1 [file genes-10-01048-s001.pdf]

Supplementary Table S1. Identified ICESXT/R391 family members with complete genome sequence available incorporated into Figure 3.

Element	Strain	Environment	Location/Year of isolation	Accessory Genes/Reported Functions	Acc. No.	Size (kb)	Reference
ICER391	*Providencia rettgeri*	Clinical	Pretoria, South Africa/1967	Km^r, Hg^r, DNA repair genes, sulphate transporter, toxin-antitoxin system, ATP-dependent Lon protease	AY090559	89	1
ICEpMERPH	*Shewanella putrefaciens*	River isolate	United Kingdom/1987	Hg^r, As^r	MH974755	110	This study
ICESXT (MO10)	*Vibrio cholerae* O139 MO10	Clinical	India/1992	Cm^r, Su^r, Tm^r, Spt^r, DNA repair genes, toxin-antitoxin system, ATP-dependent Lon protease, histidine kinase, diguanylate cyclase, deoxycytidine triphosphate deaminase	AY055428	99	2
ICER997	*Proteus mirabilis*	Clinical	Indian sub-continent/1978	B-lactamase gene, DNA repair genes, RM system, toxin-antitoxin system	KY433363	85	3
ICE*Ama*MED64	*Alteromonas macleodii* 'Aegean Sea MED64'	Superficial waters	Lebanon, Aegean Sea/2000	type I RMS	CP004848 (1194615-1274449)	80	4
ICE*Ama*IS1	*Alteromonas macleodii* Ionian Sea UM7	Marine	Ionian Sea, Uranian Basin west of Crete, Greece/2012	Heavy metal resistance, type I RMS	NC_021713	99	4
ICE*Mfu*Ind1a	*Marinomonas fungiae* JCM 18476	Mucus of the coral *Fungia echinata*	Andaman Sea, India/2010	RMS	LIQF00000000	66	5
ICE*Mfu*Ind1b	*Marinomonas fungiae* JCM 18476	Mucus of the coral *Fungia echinata*	Andaman Sea, India/2010	RMS	LIQF00000000	75	5
ICE*Mpr*Chn1	*Marinomonas profundimaris* D104	Surface sediment	Arctic Ocean/2010	RMS	AYOZ01000000	86	6
ICE*Pal*Ban1	*Providencia alcalifaciens*	Clinical	Bangladesh/1999	Cm^r, Spt^r, Su^r, Tm^r, toxin-antitoxin system, phenazine biosynthesis protein, lysine exporter, glyoxalase resistance, RMS	GQ463139	97	7

Element	Strain	Environment	Location/Year of isolation	Accessory Genes/Reported Functions	Acc. No.	Size (kb)	Reference
ICE*Pda*Spa1	*Photobacterium damselae* subsp. *piscicida* PC554.2	Marine	Spain/2001	Tc^r, heat-shock protein (dnaK), AAA ATPase, toxin-antitoxin system, ATP-dependent Lon protease ICE*Pmi*USA1	AJ870986	103	8
ICE*Pmi*Jpn1	*Proteus mirabilis*	Clinical	Japan/2006	Amx^r, Amc^r, Fox^r, Ctx^r	KT894734	93	9
ICE*Pmi*USA1	*Proteus mirabilis strain* HI4320	Clinical	USA	ATP-dependent helicase, DNA repair proteins, toxin-antitoxin system	AM942759	81	10
ICE*Spu*PO1	*Shewanella putrefaciens* W3-18-1	Marine sediment	Pacific Ocean/2000	Cu^r, Zn/Co/Cd RND efflux pump, DNA repair genes, restriction modification system	CP000503	111	11
ICE*Val*A056-1	*Vibrio alginolyticus* A056	Gill of a Litopenaeus vannamei	China/2003	Spt^r, Su^r, Az^r, type III RM system	KR231688	89	12
ICE*Val*A056-2	*Vibrio alginolyticus* A056	Gill of a Litopenaeus vannamei	China/2003	type I RM system	KR231689	104	12
ICE*Val*ASI1*	*Vibrio alginolyticus* ANC4-19	Marine	Andaman and Nicobar Islands, India/2010	L-lactate degradation system	NZ_LTYK00000000	N/A	13
ICE*Val*E0601	*Vibrio alginolyticus* E0601	Marine (Seawater)	China/2006	type III RM system	KT072768	106	12
ICE*Val*HN396	*Vibrio alginolyticus* HN396	Marine (Seawater)	China/2008	type II RMS	KT072770	87	12
ICE*Val*HN437	*Vibrio alginolyticus* HN437	Marine (Seawater)	China/2008	type I RM system	KT072771	94	12
ICE*Val*HN492	*Vibrio alginolyticus* HN492	Marine (Seawater)	China/2008	type III RM system	KT072769	106	12
ICE*Vch*Ban11	*Vibrio cholerae* 4672	Clinical	Bangladesh/2000	type II restriction system	ERS016137	96	Direct submission
ICE*Vch*Ban5	*Vibrio cholerae* O1	Clinical	Bangladesh/1998	Cm^r, Spt^r, Su^r, Tm^r, toxin-antitoxin system	GQ463140	102	7
ICE*Vch*Ban8	*Vibrio cholerae*	Clinical	Bangladesh/2001	Ac^r, Toxin-antitoxin system	JQ345361	103	7

Element	Strain	Environment	Location/Year of isolation	Accessory Genes/Reported Functions	Acc. No.	Size (kb)	Reference
	O37 strain MZ03						
ICE*Vch*Ban9	*Vibrio cholerae* MJ-1236	Clinical	Bangladesh/1994	Cm^r, Spt^r, Su^r, Tm^r, Tc^r	CP001485	108	7
ICE*Vch*Chn0143	*Vibrio cholerae* ICDC-VC0143	Clinical	China/2001	Spt^r, Su^r, Tm^r, Tc^r, toxin-antitoxin system	KT151654	87	14
ICE*Vch*Chn0956	*Vibrio cholerae* ICDC-VC956	Water	China/2001	Spt^r, Su^r, Tm^r, toxin-antitoxin system	KT151655	94	14
ICE*Vch*Chn1605	*Vibrio cholerae* ICDC-VC1605	Clinical	China/1993	Spt^r, Su^r, Tm^r, toxin-antitoxin system	KT151656	98	14
ICE*Vch*Chn1627	*Vibrio cholerae* ICDC-VC1627	Clinical	China/1997	Spt^r, Su^r, Tm^r, Tc^r, toxin-antitoxin system	KT151657	102	14
ICE*Vch*Chn1909	*Vibrio cholerae* ICDC-VC1909	Clinical	China/1998	Spt^r, Su^r, Tm^r, toxin-antitoxin system	KT151658	109	14
ICE*Vch*Chn1944	*Vibrio cholerae* ICDC-VC1944	Unknown	China/2000	Spt^r, Su^r, Tm^r, toxin-antitoxin system	KT151659	101	14
ICE*Vch*Chn2255	*Vibrio cholerae* ICDC-VC2255	Clinical	China/2008	Spt^r, Su^r, Tm^r, Tc^r, type I RM system, toxin-antitoxin system	KT151660	95	14
ICE*Vch*Chn2605	*Vibrio cholerae* ICDC-VC2605	Clinical	China/1998	Spt^r, Su^r, Tm^r, toxin-antitoxin system	KT151661	98	14
ICE*Vch*Chn*306**	*Vibrio cholerae* E306	Clinical	China/2013	toxin-antitoxin system, ATP-dependent Lon protease	NZ_AWWA01 000000	N/A	15
ICE*Vch*Chn4210	*Vibrio cholerae* ICDC-VC4210	Clinical	China/1999	Spt^r, Su^r, Tm^r, Tc^r, toxin-antitoxin system	KT151662	110	14
ICE*Vch*Chn57	*Vibrio cholerae* ICDC-VC57	Clinical	China/2005	Spt^r, Su^r, Tm^r, Tc^r, toxin-antitoxin system	KT151664	96	14
ICE*Vch*Chn1307	*Vibrio cholerae* ICDC-1307	Clinical	China	Cm^r, $Tm^{r,}$ DNA repair genes	KJ817376.1	104	Direct submission
ICE*Vch*ChnAHV1003	*Vibrio cholerae* AHV1003	Clinical	China/2010	Em^r, Spt^r, Su^r, Tm^r, type I RM system, toxin-antitoxin system	KT151663	102	14
ICE*Vch*Hai1	*Vibrio cholerae* VC1786	Clinical	Haiti/2010	Tm^r, Su^r, Spt^r, toxin-antitoxin system	JN648379	98	16
ICE*Vch*Hai2	*Vibrio cholerae* HC-1A2	Clinical	Haiti/2010	RM system	AJRO01000008	84	17
ICE*Vch*Ind4	*Vibrio cholerae* 0139	Clinical	India/1997	Cm^r, Sptr, Su^r, Toxin-antitoxin system	GQ463141	95	7

Element	Strain	Environment	Location/Year of isolation	Accessory Genes/Reported Functions	Acc. No.	Size (kb)	Reference
ICE*Vch*Ind5	*Vibrio cholerae* O1	Clinical	India/1994	Cm^r, Spt^r, Su^r, Tm^r, glyoxalase resistance, toxin-antitoxin system	GQ463142	98	7
ICE*Vch*Ind6	*Vibrio cholerae* 4605	Clinical	India/2007	Tm^r	ERS013257	90	18
ICE*Vch*Mex1	*Vibrio cholerae* non O1-0139	Sewage sample	Mexico/2001	RMS, toxin-antitoxin system	GQ463143	83	7
ICE*Vch*Moz10	*Vibrio cholerae* O1 El Tor B33	Clinical	Mozambique/2004	Spt^r, Su^r, Tc^r, toxin-antitoxin system	ACHZ00000000	104	19
ICE*Vch*B33	*Vibrio cholerae* O1 El Tor MJ123	Clinical	India/1994	Tm^r, Cm^r, Tc^r, Su^r, Sp^r, Co:Zn:Cd resistance	ACHZ00000000	106	19
ICE*Vfl*Bra1*	*Vibrio fluvialis* 560	Marine (Oyster)	Brazil/2002	RMS	JQHX00000000	N/A	20
ICE*Vfl*Bra2*	*Vibrio fluvialis* 539	Marine (Oyster)	Brazil/2002	RMS	JQHW00000000	N/A	20
ICE*Vfl*Ind1	*Vibrio fluvialis*	Clinical	India/2002	Cm^r, Sm^r, Su^r, Tm^r, toxin-antitoxin system, RMS	GQ463144	114	7
ICE*Apl*2	*Actinobacillus pleuropneumoniae MIDG3553*	Clinical	United Kingdom/2012	Sm^r, Su^r, Tm^r, DNA repair genes, RMS	MF187965.1	94	21
ICE*Vch*Thd1	*Vibrio Cholerae* TSY216	Clinical	Thailand/2010	Cm^r, Su^r, Tc^r, Tm^r, Ma^r and RMS	CP007653.1 (579719-680801)	101	22
ICE*VFlH*-08942	*Vibrio fluvialis* H08942	Clinical	India/2002	Sm^r, Cm^r, Tm^r, DNA repair genes, RMS	KM213605.1	103	23
ICE*Elba*HL53	*Idiomarinaceae bacterium,* HL-53	Unknown	Unknown	RMS	LN899469.1	69	Direct submission
ICE*Alt*Mex1	*Alteromonas sp.* Mex14	Aquaculture pond	Mexico/2014	RMS, Heavy metal resistance protein	CP018023	83	24
ICE*PSt*33672	*Providencia stuartii* ATCC 33672	Unknown	Unknown	RMS, Hg^r	CP008920.1	76	25
ICE*Val*Chn1	*Vibrio alginolyticus* ZJ-T	Marine (Epinephelus coioides)	China/2005	Fq^r, Tm^r, Su^r, Ag^r, RMS	CP016224.1	86	Direct submission
ICE*Vch*Rua1	*Vibrio Cholerae* RND68789	Clinical	Russia/2012	Bm^r, Spt^r, Su^r, DNA repair genes, abortive phage resistance protein	KY382507.1	98	26

Element	Strain	Environment	Location/Year of isolation	Accessory Genes/Reported Functions	Acc. No.	Size (kb)	Reference
ICE*Vch*Rua2	*Vibrio cholerae O1 biovar El Tor* Inaba RND18826	Clinical	Russia/2012	Bm^r, Spt^r, Su, DNA repair genes, abortive phage resistance protein	KY382506.1	98	26
ICE*Vch*Nig1	*Vibrio Cholerae* VC833	Clinical	Nigeria/2010	Spt^r, Tm^r Bm^r Su^r, Flo^r, DNA repair genes	KC886258.1	98	27
ICE*Vch*Nep1	*Vibrio Cholerae* VC504	Clinical	Nepal/1994	Spt^r, Tm^r Bm^r Su^r, DNA repair genes	KC886257.1	98	27
ICE*Pvu*CHN2213	*Proteus vulgaris* 08MAS2213	Food	China/2008	Bm ^{r} DNA repair genes, RMS Am^r, Az^r, Cm^r, Cfz^r, A/S^r, SXT^r, Su^r, Sm^r	KX243403.1	94	28
ICE*Pmi*Chn1	*Proteus mirabilis* PM13C04	Poultry	China/2012-2014	DNA repair genes, Flo^r, Tc^r, Spt^r, Su^r	KT962845.1	94	28
ICE*Pmi*Chn2	*Proteus mirabilis* JN7	Poultry	China/2013	Su^r, Bm^r ultraviolet light resistance protein B, DNA repair genes	KY437726.1	106	29
ICE*Pmi*Chn3	*Proteus mirabilis* JN28	Poultry	China/2013	Hm^r, Tm^r, Em^r, Spt^r, DNA repair genes	KY437727.1	57	29
ICE*Pmi*Chn4	*Proteus mirabilis* JN49	Poultry	China/2013	β-lactamase, DNA repair genes, Sum^r, Spt^r, Flo^r	KY437728.1	92	29
ICE*Pmi*CHN1586	*Proteus mirabilis* 08MAS1586	Food	China/2008	Tm^r, Flo$_r$, Bm^r, Spt^r, Su^r, DNA repair genes	KX243404.1	99	28
ICE*Pmi*CHN2407	*Proteus mirabilis* 09MAS2407	Clinical	China/2009	Tc^r, RMS, Co-Zn-Cd resistance, DNA repair genes, Hg^r	KX243405.1	97	28
ICE*Pmi*CHN2410	*Proteus mirabilis* 09MAS2410	Clinical	China/2009	DNA repair polymerases, RMS, Co-Zn-Cd resistance, Hg^r, Am^{r,} Azm^r, Cm^r, Km^r, Sptr, SXT^r, Su^r, Tc^r, CFZ^r, Cip^r	KX243406.1	93	28
ICE*Pmi*CHN2416	*Proteus mirabilis* 09MAS2416	Clinical	China/2009	RMS, Co-Zn-Cd resistance, DNA repair genes, Hg^r	KX243407.1	92	28
ICE*Pmi*CHN901	*Proteus mirabilis* MD20140901	Clinical	China/2014	Tm^r, Bm^r, Flo^r, Spt^r, RMS, DNA repair genes	KX243208.1	89	28
ICE*Pmi*CHN902	*Proteus mirabilis* MD20140902	Clinical	China/2014	Bm^r, Spt^r, Su^r, Am^r Azm^r, Az^r, Amk^r, Cm^r, Cip^r, Km^r, SXT^r, Tc^t, DNA repair genes, RMS	KX243409.1	89	28
ICE*Pmi*CHN903	*Proteus mirabilis MD20140903*	Clinical	China/2014	Bm^r, Tm^r, Sptr, Spt^r, RMS, DNA repair genes	KX243410.1	90	28
ICE*Pmi*CHN904	*Proteus mirabilis* MD20140904	Clinical	China/2014	Bm^r, RMS, Co-Zn-Cd resistance, DNA repair genes, Spt^r, Su^r, Tc^r	KX243411.1	95	28

Element	Strain	Environment	Location/Year of isolation	Accessory Genes/Reported Functions	Acc. No.	Size (kb)	Reference
ICE*Pmi*CHN905	*Proteus mirabilis* MD20140905	Clinical	China/2014	Bm^r, RMS, Tc^r, Spt^r, Su^r, Co-Zn-Cd resistance protein	KX243412.1	95	28
ICE*Pmi*CHN1809	*Proteus mirabilis* TJ1809	Clinical	China/2013	Am^r, Km^r, SXT^r, Azm^r, Cm^r, Su^r, Tc^r, Cf^r	KX243413.1	76	28
ICE*Pmi*CHN3237	*Proteus mirabilis* TJ3237	Clinical	China/2013	DNA repair genes, RMS, CFZ^r, Am^r, Azm^r, SXT^r, Tc^r, Su^r	KX243414.1	87	28
ICE*Pmi*CHN3300	*Proteus mirabilis* TJ3300	Clinical	China/2013	Tm^r, Azm^r, Cm^r, SXT^r, Su^r, Spt^r, DNA repair genes	KX243415.1	108	28
ICE*Pmi*CHN3335	*Proteus mirabilis TJ3335*	Clinical	China/2013	Azm^r, Bm^r, RMS, Tc^r, Spt^r, DNA repair polymerases	KX243416.1	90	28
ICE*Pmi*Chn15C1	*Proteus mirabilis Pm15C1*	Poultry	China	DNA repair genes, Tm^r, RMS	KX268685.1	65	Direct Submission
ICE*Vfl*Chn1*	*Vibrio fluvialis* 12605	Clinical (bile)	China/2013	Bile resistance genes (toxR, ompU, ompT, tolC), Heat stock proteins (GroES, GroEL, HspA), virulence factors & prophage regions	CP019118.1	N/A	Direct Submission
ICE*Vflu*Chn2*	*Vibrio fluvialis* 12605	Clinical (bile)	China/2013	Bile resistance genes (toxR, ompU, ompT, tolC), Heat stock proteins (GroES, GroEL, HspA), virulence factors & prophage regions	CP019119.1	N/A	Direct Submission
ICE*Pmi*AR_0159	*Proteus mirabilis* AR_0159	Unknown	Unknown	Cm^r, Flo^r, Tm^r, Heavy metal resistance protein.	CP021550.1	99	Direct Submission
ICE*Sup*CHN110003	*Shewanella upenei* 110003	Clinical	China/2011	DNA repair genes, Bm^r, Cm^r, Spt^r, Su^r, RMS.	MG014393.1	92	30
ICE*Pmi*Fra1	*Proteus mirabilis* PmPHI	Clinical	France/2012	Flo^r, Cm^r Spt^r, Su^r, Tc^r, Km^{r,} Tm^r, Em^r, Nm^r, RMS, merR family gene & DNA repair genes	MF490434.1	107	31
ICE*Sha*Jpn1*	*Shewanella halifaxiensis* 6JANF4-E-4	Marine (fish intestine)	Japan (Ehime, Uwa Sea)/2013	Ma^r, Flo^r, Su^r, β-lactamase	BFBQ01000001.1	N/A	32
ICE*Sha*Jpn2*	*Shewanella halifaxiensis* 6JANF4-E-4	Marine (fish intestine)	Japan (Ehime, Uwa Sea)/2013	Ma^r, Flo^r, Su^r, β-lactamase	BFBQ01000005.1	44.6	32

Element	Strain	Environment	Location/Year of isolation	Accessory Genes/Reported Functions	Acc. No.	Size (kb)	Reference
ICE*Apl*Chn1	*Actinobacillus pleuropneumoniae* App6	Animal (lung sample from a pig with respiratory disease)	China/2013	Flo^{r,} Cm^{r}, Tc, Amk^{r}, Km^{r}, Spt^{r}, Til^{r}, Em^{r}, Clr^{r}, Lm^{r}, Su^{r}	KX196444	100	33
ICE*Pmi*ChnBCP11	*Proteus mirabilis* BCP11	Diarrheic piglet	China	Tc^{r}, Km^{r}, Nm^{r}, Blm^{r}, Rif^{r}, Hym^{r}, Am^{r}, Spt^{r}, Ctx^{r}, Az^{r}, Spm^{r}, Ap^{r}, To^{r}, Tm^{r}, SXT, Cf^{r}, Nf^{r}, Fm^{r}, Cm^{r}, Flo^{r}, RMS, Nal^{r}	MG773277.1	141	34
ICE*Asp*BS1	*Alteromonas sp.* RW2A1	Seawater	Baltic Sea/2013	Calcium/sodium antiporter, RMS, multidrug efflux RND transporter, heavy metal transporter	CP018031.1 (1192908-2109146)	116	24
ICE*Vpa*Can1	*Vibrio parahaemolyticus* S107-1	Marine (Oyster)	Canada/2005	BREX (bacteriophage exclusion system), toxin HipA, XRE family transcriptional regulator	CP028481	79	35
ICE*Sh*95	*Shewanella sp. Sh95*	Clinical (ocular secretion)	Argentina/2005	*blaOXA-48*, RMS, ArsR family transcriptional regulator, zinc transporter, multidrug transporter, heavy metal transport, MerR family transcriptional regulator, cobalt transporter, dihydrofolate reductase, ethidium bromide resistance.	LGYY01000082 LGYY01000240 .1	110	36
ICE*Ama*D7	*Alteromonas macleodii, D7*	Marine (seawater)	Thailand (Andaman Sea)/2000	Hg^{r}, Ar^{r}, Cu^{r}, Ca proton antiporter, mechanosensitive ion channel, RMS, multidrug transporter, hydrophobe/amphiphile efflux protein,	CP014323 (1261745-1375908)	114	Direct submission
ICE*Eco*HVH177	*Escherichia coli* HVH 177	Clinical	Denmark/2003	Unknown	AZJM01000017	97	Direct submission
ICE*Mfr*JAM7	*Methylophaga frappieri* JAM7	Marine (Seawater)	Canada/Unknown	RMS, toxin-antitoxin system, molybdenum cofactor biosynthesis enzyme, multidrug resistance efflux pump	CP003380 (495596-606053)	110	37
ICE*Sde*ChnS12	*Shewanella decolorationis* S12	Wastewater treatment plant	China/2012	AAA ATPase, RMS	AXZL01000060 .1	71	38
ICE*Val*ZJT1	*Vibrio alginolyticus* ZJ-T	Marine (Orange-spotted grouper)	China/2005	Fq^{r}, Km^{r}, Tm^{r}, RMS	CP016224 (2476706-2564476)	89	39

Element	Strain	Environment	Location/Year of isolation	Accessory Genes/Reported Functions	Acc. No.	Size (kb)	Reference
ICE*Val*ZJT2	*Vibrio alginolyticus* ZJ-T	Marine (Orange-spotted grouper)	China/2005	RMS, Calcium: sodium antiporter	CP016224 (936462-1036489)	100	39
ICE*Vch*2012HC25**	*Vibrio cholerae* O1 El Tor 2012HC-25	Clinical	Haiti/2012	NA	JSTY01000047	77	40
ICE*Vch*2012Env25**	*Vibrio cholerae* non-O1/O139 2012Env-25	Water	Haiti/2012	NA	JSTE01000047	108	40
ICE*Vch*8-76-1**	*Vibrio cholerae* O77 8-76-1	Clinical	India/1976	NA	JIDN01000032	102	41
ICE*Vch*8-76-2**	*Vibrio cholerae* O77 8-76-2	Clinical	India/1976	NA	JIDN01000012	122	41
ICE*Vch*YB8E08**	*Vibrio cholerae* YB8E08	Marine (Oyster pond)	USA/2009	NA	LBGN01000012	104	42
ICE*Vch*YP6E07	*Vibrio cholerae* OYP6E07	Marine (Oyster pond)	USA/2009	RMS, heavy metal resistance, AAA family ATPase	NMTB0100001 4	93	Direct submission
ICE*Vch*3272-78	*Vibrio cholerae* 3272-78	Water	USA/1977	Ac^r, Tc^r, Multidrug transporter, transcriptional regulator, ToxR.	MIOZ01000052	104	Direct submission
ICE*Vch*3223-74	*Vibrio cholerae* 3223-74	Storm drain	Guam/1974	Tc^r, Ac^r, transcriptional regulator ToxR, AraC family transcriptional regulator	MIZG01000083	104	Direct submission
ICE*Vmy*CAIM528**	*Vibrio mytili* CAIM528	Seawater	Spain/1985	RMA, abortive phage resistance system, XRE family transcriptional regulator	JXOK01000005	67	Direct submission
ICE*Vpa*Chn25	*Vibrio parahaemolyticus* CHN25	Marine (Shrimps)	China/2011	Su^r, Sm^r, Tc^r, Tm^r, RMS	CP010883	88	43
ICE*Vpa*S163**	*Vibrio parahaemolyticus* S163	Marine (Seafood)	Malaysia/2007	NA	AWHQ0100000 4	76	44
ICE*Vpa*S167**	*Vibrio parahaemolyticus* S167	Environment	China/2007	NA	AWHM010000 24	82	44
ICE*Vpa*UCM493	*Vibrio parahaemolyticus* UCM-V493	Sediment	Spain/2002	Beta-lactamase, RMS, transcriptional regulator, XRE, Heat stock proteins	CP007004	111	45

Element	Strain	Environment	Location/Year of isolation	Accessory Genes/Reported Functions	Acc. No.	Size (kb)	Reference
ICE*Vvu*SC9729**	*Vibrio vulnificus* SC9729	Seawater	South Korea/2011	Cobalt import ATP binding protein	JZEQ01000086	85	Direct submission
ICE*Vvu*CladeA158**	*Vibrio vulnificus* CladeA-yb158	Tilapia Fish	Israel/2005	AraJ, RMS, XRE family transcriptional regulator.	LBNN01000013	89	46
ICE*Vvu*CG100**	*Vibrio vulnificus* CG100	Oyster	Taiwan/1993	AraJ, LysR family transcriptional regulator, MFS transporter	PDGD01000031	105	47
ICE*Vpa*Mal1	*Vibrio parahaemolyticus* PCV08-7	Seafood	Malaysia/2008	As^r, RMS	NZ_AOCL01000005	71	48
ICE*Vpa*Mal2	*Vibrio parahaemolyticus* ND24	Marine	Malaysia/2017	As^r, RMS	NZ_POAW01000003	79	Direct submission
ICE*Vpa*Mal3	*Vibrio parahaemolyticus* ND22	Marine	Malaysia/2017	As^r, RMS	NZ_POAY01000002	77	Direct submission
ICE*Vpa*Mal4	*Vibrio parahaemolyticus* ND19	Marine	Malaysia/2017	As^r, RMS	NZ_POBB01000003	77	Direct submission
ICE*Vpa*Mal5	*Vibrio parahaemolyticus* ND16	Marine	Malaysia/2017	As^r, RMS	NZ_POBE01000002	77	Direct submission
ICE*Vpa*Mal6	*Vibrio parahaemolyticus* ND13	Marine	Malaysia/2017	As^r, RMS	NZ_POBH01000002	77	Direct submission
ICE*Vpa*Mal7	*Vibrio parahaemolyticus* ND12	Marine	Malaysia/2017	As^r, RMS	NZ_POBI01000004	77	Direct submission
ICE*Vpa*Mal8	*Vibrio parahaemolyticus* ND11	Marine	Malaysia/2017	As^r, RMS	NZ_POBJ01000003	77	Direct submission
ICE*Vpa*Mal9	*Vibrio parahaemolyticus* NA1	Marine	Malaysia/2017	As^r, RMS	NZ_POBW01000001	77	Direct submission

Element	Strain	Environment	Location/Year of isolation	Accessory Genes/Reported Functions	Acc. No.	Size (kb)	Reference
ICE*Vpa*Mal10	*Vibrio parahaemolyticus* NA2	Marine	Malaysia/2017	As^r, RMS	NZ_RQNT01000003	77	Direct submission
ICE*Vpa*Mal11	*Vibrio parahaemolyticus* ST17.P5-S1	Marine	Malaysia/2017	As^r, RMS	NZ_PJOR01000002	77	Direct submission
ICE*Vpa*Thd1	*Vibrio parahaemolyticus* NCKU_TV_3HP	Marine	Thailand/1999	As^r, RMS	NZ_JPKS01000041	79	49
ICE*Vpa*Thd2	*Vibrio parahaemolyticus* NCKU_TV_5HP	Marine	Thailand/1999	As^r, RMS	NZ_JPKT01000008	77	49
ICE*Vpa*Vie01	*Vibrio parahaemolyticus* M1-1	Marine	Vietnam/2014	As^r, RMS	NZ_PDDQ01000009	79	50

SXT/R391 ICEs with ** found in the study by (Bioteau *et al*. 2018), ICEs are scattered over two contigs extracted WGS data, an estimation of the minimum size is provided.

*Unpublished Sequence data ** Not annotated or partially annotated

Abbreviations associated with this table: AAA: ATPases Associated with diverse cellular activities **, Ac^r** : Acriflavin resistance , **Af^r**: Actiflavine resistance**, Ag^r**: Aminoglycoside resistance, **Am^r**: Ampicillin resistance, **Amk^r** :Amikacin resistance**, Ap^r**: Apramycin resistance, **As^r:** Arsenic resistance, **Azm^r:** Azithromycin resistance, **Az^r**: Aztreonam resistance, **A/S^r**: Ampicillin-sulbactam resistance, **Bm^r**- Bicyclomycin resistance, **Blm^r**: Bleomycin resistance, **Co**: Cobalt**, Cd**: Cadmium **, Cfz^r:** Cefazolin resistance **Ctx^r**: Cefotaxime resistance**, Cip^r**: Ciprofloxacin resistance**, Cli^r**: Clindamycin resistance**, Cm^r**: Chloramphenicol resistance, **Cu^R**: Copper resistance, **Em^r**: Erythromycin resistance, **Flo^r**: Florfenicol resistance, **Fm^r**: Fosfomycin resistance, **Fq^r**: Fluoroquinolone resistance, **Hg^r**: Mercury resistance, **Hm^r**: Hygromycin resistance, **Km^r**: Kanamycin resistance, **Lm^r**: lincomycin resistance, **Ma^r**: Macrolide resistance, **Nal^r**: Nalidixic acid resistance, **Nm^r**: Neomycin resistance, **Nf^r**: Norfloxacin resistance, **Rif^r**: Rifampicin resistance, RND: Resistance Nodulation Cell Division, RMS: Restriction modification system, Spt^r: Spectinomycin resistance, Sm^r: Streptomycin resistance, **Sum^r:** Sulphonamide resistance, **Su^r**: Sulfamethoxazole resistance, **SXT**: Sulfamethoxazole: Trimethoprim resistance, **Til^r**: Tilmicosin resistance**, Tc^r**: Tetracycline resistance, **Tm^r**: Trimethoprim resistance, **To^r**: Tobramycin resistance, **Zn**: Zinc. **NA**: Not Available

Supplementary Table S2. Disk diffusion test results, results determined by reference to the EUCAST Clinical Breakpoint tables for Enterobacterales.

Antibiotic	AB1157, Zone diameter (mm)	Result	AB1157: pMERPH, Zone diameter (mm)	Result
**Penicillin's**				
Amoxycillin/clavulanic acid (20/10 µg)	20	Susceptible	19	Susceptible
Ampicillin (10 µg)	20	Susceptible	19	Susceptible
Piperacillin (36 µg)	38	Susceptible	29	Susceptible
Piperacillin/tazobactam 30/6 µg)	19	Intermediate	19	Intermediate
Ticarcillin/clavulanic acid, 75/10 µg)	29	Susceptible	25	Susceptible
**Cephalosporins**				
Cefoxitin (30 µg)	30	Susceptible	20	Susceptible
Cefpodoxime (10 µg)	32	Susceptible	29	Susceptible
Ceftazidime (10 µg)	24	Susceptible	24	Susceptible
Ceftiofur (30 µg)	20	Susceptible	16	Intermediate
Cephalothin (30 µg)	20	Susceptible	17	Intermediate
**Carbapenems**				
Meropenem (10 µg)	35	Susceptible	39	Susceptible
**Monobactams**				
Aztreonam (30 µg)	33	Susceptible	30	Susceptible
**Fluoroquinolones**				
Ciprofloxacin (5 µg)	29	Susceptible	30	Susceptible
Nalidixic acid (30 µg)	25	Susceptible	25	Susceptible
Norfloxacin (10 µg)	15	Susceptible	26	Susceptible
Ofloxacin (5 µg)	32	Susceptible	29	Susceptible
**Aminoglycosides**				
Amikacin (30 µg)	23	Susceptible	22	Susceptible
Gentamin (10 µg)	21	Susceptible	20	Susceptible
Erythromycin (15 µg)	0	Resistant	0	Resistant
Kanamycin (30 µg)	23	Susceptible	19	Susceptible
Neomycin (30 µg)	21	Susceptible	19	Susceptible
**Tetracyclines**				
Tetracycline (30 µg)	30	Susceptible	29	Susceptible
Minocycline (30 µg)	24	Susceptible	24	Susceptible
**Miscellaneous agents**				
Nitrofurantoin (100 µg)	22	Susceptible	23	Susceptible
SXT (1.25/23.75 µg)	31	Susceptible	28	Susceptible
Trimethoprim (5 µg)	22	Susceptible	28	Susceptible

Supplementary Table S3. Genes of ICEpMERPH.

pMERPH	Similarity to R391	R391 equivalent	Similarity to SXT	SXT equivalent	Core/ Hotspot/ Variable Region	Putative role
**ORF 1**	99 %	Hypothetical protein (AAM08068.1)	Absent	Absent	Variable Region 1	Transcriptional regulator, DNA binding
**ORF 2**	97 %	Hypothetical protein (AAM08010.1)	Absent	Absent	Variable Region 1	Serine/threonine-protein kinase
**ORF 3**	100 %	Hypothetical protein (AAM08069.1)	Absent	Absent	Core	excision
**ORF 4**	99 %	Int (AAM08088.1)	99 %	Integrase (AAL59748.1)	Core	DNA Integration
**ORF 5**	87 %	Hypothetical protein (AAM08070.1)	91 %	s002 (AAL59745.1)	Core	Unknown
**ORF 6**	98 %	Hypothetical protein (AAM08024.1)	98 %	s003 (AAL59744.1)	Core	Rod shape determination protein
**ORF 7**	Absent	Absent	Absent	Absent		Unknown
**ORF 8**	Absent	Absent	Absent	Absent		Unknown
**ORF 9**	Absent	Absent	Absent	Absent		Undecaprenyl-diphosphatase (EC 3.6.1.27)
**ORF 10**	Absent	Absent	Absent	Absent		Inner membrane protein YrbG, predicted calcium/sodium: proton antiporter
**ORF 11**	Absent	Absent	Absent	Absent		Small-conductance mechanosensitive channel
**ORF 12**	Absent	Absent	Absent	Absent		Thiamin biosynthesis lipoprotein ApbE
**ORF 13**	Absent	Absent	Absent	Absent		Magnesium and cobalt transport protein CorA
**ORF 14**	Absent	Absent	Absent	Absent		UPF0104 membrane protein AF_2231
**ORF 15**	Absent	Absent	Absent	Absent		Glycosyltransferase
**ORF 16**	97 %	Rum B (AAM08023.1)	91 %	Rum B (AAL59747.1)	Core	DNA repair
**ORF 17**	97 %	Rum A (AAM08055.1)	96 %	Rum A (AAL59746.1)	Core	DNA binding
**ORF 18**	97 %	Hypothetical protein (AAM08020.1)	98 %	s024 (AAL59738.1)	Core	Nucleic acid binding
**ORF 19**	Absent	Absent	Absent	Absent	Hotspot 5	Unknown
**ORF 20**	Absent	Absent	Absent	Absent	Hotspot 5	Unknown
**ORF 21**	91 %	Hypothetical protein (AAM08074.1)	91 %	s025 (AAL59737.1)	Hotspot 5	Unknown
**ORF 22**	60 %	Hypothetical protein (AAM08019.1)	60 %	s026 (AAL59736.1)	Hotspot 5	Unknown
**ORF 23**	Absent	Absent	Absent	Absent	Hotspot 5	RMS, methyltransferase activity
**ORF 24**	Absent	Absent	Absent	Absent	Hotspot 5	RMS, DNA modification and binding
**ORF 25**	Absent	Absent	Absent	Absent	Hotspot 5	Anticodon nuclease, Unknown
**ORF 26**	Absent	Absent	Absent	Absent	Hotspot 5	RMS, DNA modification
**ORF 27**	Absent	Absent	Absent	Absent	Hotspot 5	Unknown
**ORF 28**	93 %	TraI (AAM08003.1)	93 %	TraI (AAL59675.1)	Core	Conjugative Transfer
**ORF 29**	99 %	TraD (AAM08004.1)	99 %	TraD (AAL59680.1)	Core	Conjugative transfer
**ORF 30**	96 %	Hypothetical protein (AAM08047.1)	Absent	Absent		Unknown

**ORF 31**	99 %	Hypothetical protein (AAM08039.1)	99 %	s043 (AAL59721.1)	Core	Unknown
**ORF 32**	Absent	Absent	Absent	Absent	Hotspot 1	DNA integration
**ORF 33**	Absent	Absent	Absent	Absent	Hotspot 1	Unknown
**ORF 34**	Absent	Absent	Absent	Absent	Hotspot 1	Unknown
**ORF 35**	Absent	Absent	Absent	Absent	Hotspot 1	Unknown
**ORF 36**	Absent	Absent	Absent	Absent	Hotspot 1	Unknown
**ORF 37**	Absent	Absent	Absent	Absent	Hotspot 1	Unknown
**ORF 38**	Absent	Absent	Absent	Absent	Hotspot 1	Unknown
**ORF 39**	Absent	Absent	Absent	Absent	Hotspot 1	DNA binding
**ORF 40**	Absent	Absent	Absent	Absent	Hotspot 1	Transporter activity
**ORF 41**	Absent	Absent	Absent	Absent	Hotspot 1	Transmembrane transport activity
**ORF 42**	Absent	Absent	Absent	Absent	Hotspot 1	Unknown
**ORF 43**	Absent	Absent	Absent	Absent	Hotspot 1	Unknown
**ORF 44**	Absent	Absent	Absent	Absent	Hotspot 1	Unknown
**ORF 45**	Absent	Absent	Absent	Absent	Hotspot 1	Unknown
**ORF 46**	Absent	Absent	Absent	Absent	Hotspot 1	ATP binding
**ORF 47**	95 %	Hypothetical protein (AAM08045.1)	Absent	Absent		Unknown
**ORF 48**	97 %	TraL (AAM08076.1)	100 %	TraL (AAL59674.1)	Core	Conjugation
**ORF 49**	99 %	TraE (AAM08060.1)	100 %	TraE (AAL59679.1)	Core	Conjugation
**ORF 50**	95 %	TraK (AAM08021.1)	95 %	s048 (AAL59718.1)	Core	Transfer
**ORF 51**	99 %	TraB (AAM08009.1)	98 %	TraB (AAL59682.1)	Core	Type IV secretion system
**ORF 52**	98 %	HtdD (AAM08037.1)	98 %	TraV (AAL59672.1)	Core	Type IV conjugative transfer system
**ORF 53**	99 %	TraA (AAM08062.1)	100 %	TraA (AAL59683.1)	Core	Type IV conjugative transfer system
**ORF 54**	Absent	Absent	Absent	Absent	Hotspot 2	N-acetyltransferase
**ORF 55**	98 %	Unknown (AAM08078.1)	Absent	Absent	Hotspot 2	Vibrio phage ICP1, regulation of transcription
**ORF 56**	Absent	Absent	Absent	Absent	Hotspot 2	Unknown
**ORF 57**	Absent	Absent	Absent	Absent	Hotspot 2	Unknown
**ORF 58**	40 %	Hypothetical protein (AAM07997.1)	Absent	Absent	Core	Abi-like family protein
**ORF 59**	98 %	DsbC (AAM08034.1)	98 %	s054 (AAL59715.1)	Core	Disulphide bond isomerase
**ORF 60**	Absent	Absent	Absent	Absent		Transposase
**ORF 61**	98 %	TraC (AAM08001.1)	99 %	TraC (AAL59681.1)	Core	Type IV secretion protein
**ORF 62**	Absent	Absent	Absent	Absent		Transposase
**ORF 63**	Absent	Absent	Absent	Absent		Unknown
**ORF 64**	99 %	TrhF (AAM08054.1)	96 %	TrsF (AAL59670.1)	Core	Conjugative signal peptidase
**ORF 65**	95 %	TraW (AAM08011.1)	95 %	TraW (AAL59671.1)	Core	Type-F conjugative transfer system
**ORF 66**	98 %	TraU (AAM08014.1)	99 %	TraU (AAL59673.1)	Core	Conjugal transfer protein
**ORF 67**	98 %	TraN (AAM07995.1)	98 %	TraN (AAL59755.1)	Core	Mating pair stabilization
**ORF 68**	Absent	Absent	Absent	Absent	Hotspot 4	Major facilitator superfamily
**ORF 69**	Absent	Absent	Absent	Absent	Hotspot 4	Glyceraldehyde 3-phosphate dehydrogenase
**ORF 70**	Absent	Absent	Absent	Absent	Hotspot 4	Protein phosphatase Dephosphorylation
**ORF 71**	Absent	Absent	Absent	Absent	Hotspot 4	Thioredoxin family protein
**ORF 72**	Absent	Absent	Absent	Absent	Hotspot 4	Permease
**ORF 73**	Absent	Absent	Absent	Absent	Hotspot 4	ACR3 family arsenite efflux transporter
**ORF 74**	Absent	Absent	Absent	Absent	Hotspot 4	Metalloregulator ArsR family transcription factor

**ORF 75**	98 %	Hypothetical protein (AAM08043.1)	100 %	s063 (AAL59711.1)	Core	Unknown
**ORF 76**	96 %	Hypothetical protein (AAM08064.1)	Absent	Absent	Core	Plasmid-related protein
**ORF 77**	97 %	Hypothetical protein (AAM08058.1)	95 %	Ssb (AAL59691.1)	Core	Single-stranded DNA-binding protein
**ORF 78**	100 %	Hypothetical protein (AAM08027.1)	99 %	s065 (AAL59710.1)	Core	Phage recombination protein BET
**ORF 79**	Absent	Absent	Absent	Absent		Unknown
**ORF 80**	97 %	Hypothetical protein (AAM08015.1)	98 %	s066 (AAL59709.1)	Core	Endonuclease
**ORF 81**	98 %	Hypothetical protein (AAM08012.1)	98 %	s067 (AAL59708.1)	Core	Aerobic cobaltochelatase CobS
**ORF 82**	97 %	Hypothetical protein (AAM08029.1)	Absent	Absent		Unknown
**ORF 83**	99 %	Hypothetical protein (AAM08016.1)	99 %	s068 (AAL59707.1)	Core	Unknown
**ORF 84**	96 %	Unknown (AAM08081.1)	97 %	s069 (AAL59706.1)	Core	Unknown
**ORF 85**	99 %	Hypothetical protein (AAM08006.1)	98 %	s070 (AAL59705.1)	Core	Plasmid associated gene product
**ORF 86**	98 %	Hypothetical protein (AAM08053.1)	98 %	s071 (AAL59704.1)	Core	DNA repair protein RadC
**ORF 87**	92 %	Hypothetical Protein (AAM08083.1)	Absent	Absent	Core	Plasmid-related protein
**ORF 88**	97 %	Hypothetical Protein (AAM08013.1)	98 %	s072 (AAL59703.1)	Core	Zinc binding domain protein
**ORF 89**	84 %	Hypothetical Protein (AAM08033.1)	83 %	s073 (AAL59702.1)	Core	Unknown
**ORF 90**	Absent	Absent	Absent	Absent	Hotspot 3	Restriction endonuclease
**ORF 91**	Absent	Absent	Absent	Absent	Hotspot 3	ISL3 family transposase
**ORF 92**	Absent	Absent	Absent	Absent	Hotspot 3	ArsR family transcriptional regulator
**ORF 93**	Absent	Absent	Absent	Absent	Hotspot 3	Permease
**ORF 94**	Absent	Absent	Absent	Absent	Hotspot 3	Restriction endonuclease
**ORF 95**	Absent	Absent	Absent	Absent	Hotspot 3	McrBC 5-methylcytosine restriction system protein
**ORF 96**	95 %	TraF (AAM08018.1)	95 %	TraF (AAL59678.1)	Core	Thioredoxin family protein/ Type IV conjugative transfer system
**ORF 97**	98 %	TraH (AAM08008.1)	99 %	TraH (AAL59676.1)	Core	Type IV conjugative transfer system protein
**ORF 98**	99 %	TraG (AAM07996.1)	98 %	TraG (AAL59677.1)	Core	Conjugal transfer
**ORF 99**	100 %	MerR (AAM08061.1)	Absent	Absent	Variable Region 4	Mercury (II) responsive transcriptional regulator
**ORF 100**	98 %	Mer T (AAM08065.1)	Absent	Absent	Variable Region 4	Mercury transporter
**ORF 101**	100 %	Mer P (AAM08084.1)	Absent	Absent	Variable Region 4	Mercury resistance system periplasmic binding
**ORF 102**	98 %	Mer C (AAM08059.1)	Absent	Absent	Variable Region 4	Mercury ion transport
**ORF 103**	100 %	Mer A (AAM08005.1)	Absent	Absent	Variable Region 4	Mercury reductase activity
**ORF 104**	98%	Hypothetical Protein (AAM08057.1)	71 %	s079 (AAL59699.1)	Core	Exclusion system protein Eex

**ORF 105**	98 %	Hypothetical Protein (AAM08050.1)	99 %	SetC (AAL59693.1)	Core	Flagellar Transcriptional regulator FlhC
**ORF 106**	100 %	Hypothetical protein (AAM08085.1)	100 %	SetD (AAL67891.1)	Core	Flagellar transcriptional activator FlhD
**ORF 107**	98 %	Hypothetical Protein (AAM08048.1)	98 %	s082 (AAL59698.1)	Core	Lytic transglycosylase domain-containing protein
**ORF 108**	99 %	Hypothetical Protein (AAM08041.1)	97 %	s083 (AAL59697.1)	Core	Unknown
**ORF 109**	98 %	Hypothetical Protein (AAM08022.1)	98 %	s085 (AAL59695.1)	Core	Unknown
**ORF 110**	46 %	Putative transcriptional regulator (AAM08038.1)	98 %	s086 (AAL59694.1)	Core	Cro/Cl family transcriptional regulator
**ORF 111**	100 %	Putative transcriptional regulator (AAM08038.1)	100 %	SetR (AAL59692.1)	Core	LexA family transcriptional regulator
**ORF 112**	Absent	Absent	Absent	Absent		PrfC (peptide chain release factor 3)

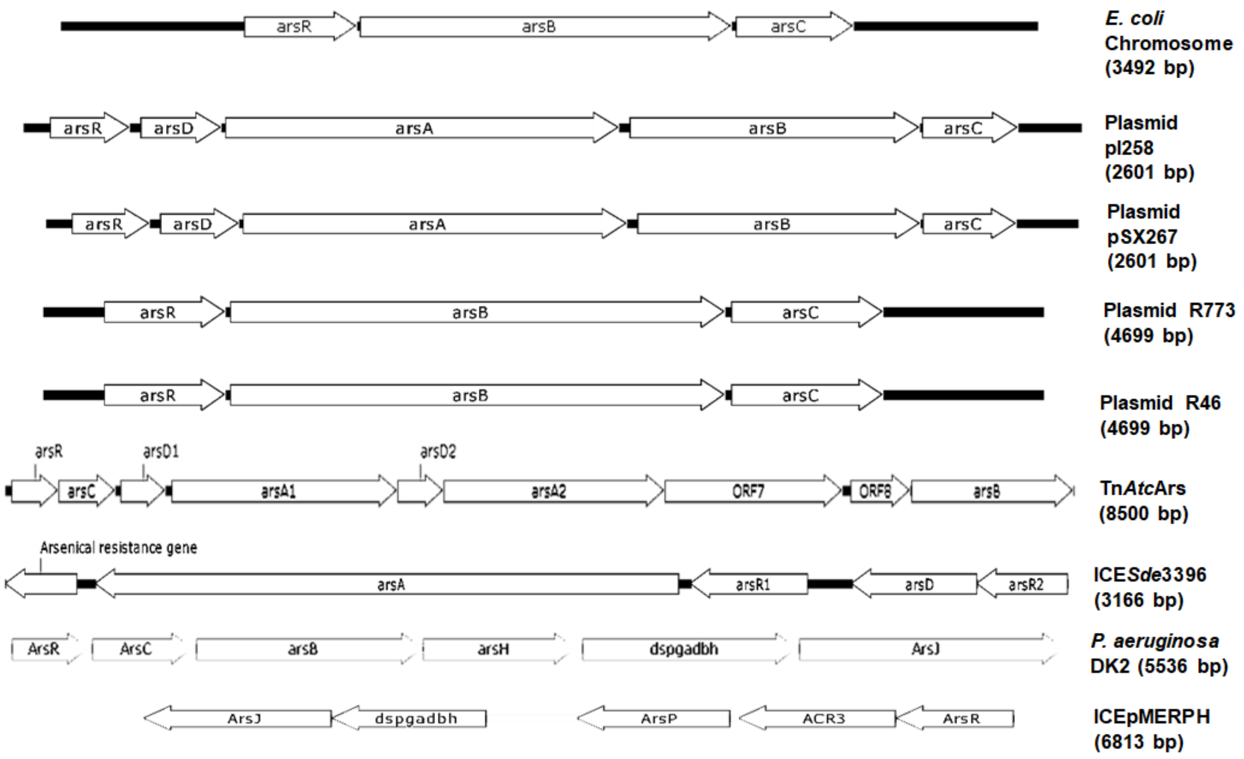

Supplementary Figure S1. Common arsenic resistant systems located on the *E. coli* chromosome, on *E. coli* plasmids R773 and R46 and on Staphylococcus aureus plasmids pI258 and pSX267, a novel arsenic resistant system found on the transposon TnAtcArs, the ICE mobile genetic ICESde3369, the resistant system found on the chromosome in *P. aeruginosa* DK2 and the arsenic resistant system found in ICEpMERPH.

Comparative analysis was carried out to compare the arsenic resistant system found in HS4 of ICEpMERPH with other well studied arsenic resistant systems. This should give an insight into how this system differs from other well-studied arsenic resistant systems. Well studied arsenic systems are found on the chromosome of certain *Escherichia coli* and in bacterial mobile genetic elements. All these systems have similar molecular gene structure, the *E. coli* encoded arsenic operon contains genes *arsRBC* [51] and the same arrangement is observed in both plasmids contained in gram-positive *Staphylococcus aureus* pI258 (RBC) [52] and *Staphylococcus xylosus* pSX267 (RBC) [53].

Similarly, the plasmids R773 and R46 share three of the same genes with the addition of *arsA* and *arsD* [54,55]. *arsA* and *arsD* are missing from the *E. coli* operon and the Staphylococcus arsenic operons. ArsD is a secondary regulator and has apparently no effect on the level of resistance. *arsA* is more important, it's a membrane transporter that can switch energy coupling modes from chemiosmotic or an ATPase pump [56].

All these well studied arsenic operons are mapped in Figure S1. The number of genes can vary between the gram-negative and gram-positive organisms and the details of their functions can also vary. R773 and R46 protein products share 85 % and 93% similarity [56] while those associated with pI258 and pSX267 share 93 % amino acid identity [56]. Plasmid associated arsenic resistant systems have been well studied but there is little information on systems in other MGE or ICEs which may have changed or evolved.

An unusual novel arsenic resistant transposon, Tn*Atc*Ars, was identified in the bacterium *Acidithiobacillus caldus* [57], its gene arrangement is different from any other system identified containing Ars RCDADA [57]. Another novel arsenic resistance system was also identified in an ICE, ICE*Sde*3369 (EU142041) which was unlike other arsenic resistance systems so far discovered [58] (Figure S1).

The predicted ICEpMERPH arsenic resistance system contained in HS4 contains 7 genes, the last three genes encoding homologs of ArsP, ACR3 and ArsR. The ArsP-like proteins are permeases similar to a methylarsenite efflux permease found in *Camylobacter jejuni* [59]. This was the first identified efflux system specific for trivalent organarsenicals [59]. The putative protein ACR3 is a homolog of an arsenite transporter that demonstrated resistance to arsenate but not arsenite in arsenic hypersensitive *E. coli* AW3110 [60]. The putative ArsR protein is a homolog of an arsenic inducible repressor [61] that is commonly seen in arsenic resistance determinants. This system is missing an arsenic reductase, and thus it does not seem to be a complete operon and therefore may not be fully functional compared to full operon containing systems and may have changed by insertion or deletion of genes over time. Putative tyrosine phosphate and thioredoxin protein encoding genes appear before these putative Ars genes but there is as yet no evidence that they have any relation to arsenic resistance.

It may be an adaptive advantage that this putative system is missing ArsC, the arsenate reductase enzyme. Arsenate reductases reduces the less toxic arsenate (As(III)) to the more toxic arsenite (As(III)) [52] where conversion of a less toxic compound to a more toxic compound seems counter-productive [56]. This may explain the two-gene system at the beginning of the hotspot, similarly found on the chromosome of *Pseudomonas aeruginosa* DK2 which confers resistance to arsenate. Detoxification systems like this may have evolved early when arsenic was reduced to As(III). This system does not appear to require cellular reductants or reductases and it does not require biotransformation to more reactive and toxic trivalent species [62] which is a major adaptive advantage.

The ICEpMERPH system and that found in *P. aeruginosa* DK2 are illustrated in Figure S1. Which illustrates that the two systems are different in gene synteny and orientation. *P. aeruginosa* DK2 contains known arsenic resistant genes such as *arsR*, *arsC*, *acr3* and *arsH* which are different to the system found in ICEpMERPH, the only similarity being the two-gene system that confers resistance to arsenate.

Thus, we hypothesise that the ICEpMERPH system is a novel arsenate efflux system not seen in other SXT/R391 ICE's or in any other bacterial mobile element to the best of our knowledge.

References

1. Boltner, D.; MacMahon, C.; Pembroke, J.T.; Strike, P.; Osborn, A.M. R391: A conjugative integrating mosaic comprised of phage, plasmid, and transposon elements, *J. Bacteriol.* **2002**, *18*, 5158–5169, doi.org/10.1128/jb.184.18.5158-5169.2002
2. Beaber, J.W.; Hochhut, B.; Waldor, M.K. Genomic and functional analyses of SXT, an integrating antibiotic resistance gene transfer element derived from *Vibrio cholerae*. *J. Bacteriol.*, **2002**, *15*, 4259–4269.
3. Ryan, M.P.; Armshaw, P.; O'Halloran, J.A.; Pembroke, J.T. Analysis and comparative genomics of R997, the first SXT/R391 integrative and conjugative element (ICE) of the Indian Sub-Continent. *Sci. Rep*. **2007**, *7*, doi.org/10.1038/s41598-017-08735-y.
4. López-Pérez, M.; Gonzaga, A.; Rodriguez-Valera, F. Genomic diversity of "deep ecotype" Alteromonas macleodii isolates: evidence for Pan-Mediterranean clonal frames. *Genome Biol. Evol.* **2013**, *6*, 1220–1232.
5. Badhai, J.; Das, S.K. Characterization of three novel SXT/R391 integrating conjugative elements ICEMfuInd1a and ICEMfuInd1b, and ICEMprChn1 identified in the genomes of *Marinomonas fungiae* JCM 18476T and *Marinomonas profundimaris* strain D104. *Front. Microbiol.* **2016**, *7*, 1896.
6. Dong, C.; Bai, X.; Lai, Q.; Xie, Y.; Chen, X.; Shao, Z. Draft genome sequence of Marinomonas sp. strain D104, a polycyclic aromatic hydrocarbon-degrading bacterium from the deep-sea sediment of the Arctic Ocean. *Genome Announc*. **2014**, *1*, e01211-13.
7. Wozniak, R.A.F.; Fouts, D.E.; Spagnoletti, M.; Colombo, M.M.; Ceccarelli, D.; Garriss, G.; Dery, C.; Burrus, V.; Waldor, M.K. Comparative ICE Genomics: Insights into the Evolution of the SXT/R391 Family of ICEs. *PLoS Genetics*, **2009**, *12*, doi.org/10.1371/journal.pgen.1000786.
8. Osorio, C.R.; Marrero, J.; Wozniak, R.A.F.; Lemos, M.L.; Burrus, V.; Waldor, M.K. Genomic and functional analysis of ICEPdaSpa1, a fish-pathogen-derived SXT-related integrating conjugative element that can mobilize a virulence plasmid. *J. Bacteriol.* **2008**, *9*, 3353–3361, doi.org/10.1128/jb.00109-08.
9. Lei, C.-W.; Zhang, A.-Y.; Wang, H.-N.; Liu, B.-H.; Yang, L.-Q.; Yang, Y.-Q. Characterization of SXT/R391 integrative and conjugative elements in *Proteus mirabilis* isolates from food-producing animals in China. *Antimicrob. Agents Chemoth.* **2016**, *3*, 1935–1938.
10. Pearson, M.M.; Sebaihia, M.; Churcher, C.; Quail, M.A.; Seshasayee, A.S.; Luscombe, N.M.; Abdellah, Z.; Arrosmith, C.; Atkin, B.; Chillingworth, T.; et al. Complete genome sequence of uropathogenic *Proteus mirabilis*, a master of both adherence and motility. *J. Bacteriol.* **2008**, *11*, 4027–4037.
11. Pembroke, J.T.; Piterina, A.V. A novel ICE in the genome of *Shewanella putrefaciens* W3-18-1: Comparison with the SXT/R391 ICE-like elements. *FEMS Microbiol. Lett.* **2006**, *1*, 80–88.
12. Luo, P.; He, X.; Wang, Y.; Liu, Q.; Hu, C. Comparative genomic analysis of six new-found integrative conjugative elements (ICEs) in *Vibrio alginolyticus*. *BMC Microbiol.* **2016**, *1*, 79.
13. Bhotra, T.; Singh, D.V. Whole-genome sequence of *Vibrio alginolyticus* isolated from the mucus of the coral *Fungia danai* in the Andaman Sea, India', *Genome Announc.* **2016**, *3*, e00339-16.
14. Wang, R.B.; Yu, D.; Yue, J.J.; Kan, B. Variations in SXT elements in epidemic *Vibrio cholerae* O1 El Tor strains in China. *Sci. Rep.* **2016**, *6*, doi.org/10.1038/srep22733.
15. Yi, Y.; Lu, N.; Liu, F.; Li, J.; Zhang, R.; Jia, L.; Jing, H.; Xia, H.; Yang, Y.; Zhu, B.; et al. Genome sequence and comparative analysis of a *Vibrio cholerae* O139 strain E306 isolated from a cholera case in China. *Gut Pathog.* **2014**, *1*, 3.
16. Sjölund-Karlsson, M.; Reimer, A.; Folster, J.P.; Walker, M.; Dahourou, G.A.; Batra, D.G.; Martin, I.; Joyce, K.; Parsons, M.B.; Boncy, J.; et al. Drug-resistance mechanisms in *Vibrio cholerae* O1 outbreak strain, Haiti, 2010. *Emerg. Infect. Dis.* **2011**, *11*, 2151.
17. Ceccarelli, D.; Spagnoletti, M.; Hasan, N.A.; Lansing, S.; Huq, A.; Colwell, R.R. A new integrative conjugative element detected in Haitian isolates of *Vibrio cholerae* non-O1/non-O139. *Res. Microbiol.* **2013**, *9*, 891–893.
18. Chowdhury, F.; Mather, A.E.; Begum, Y.A.; Asaduzzaman, M.; Baby, N.; Sharmin, S.; Biswas, R.; Uddin, M.I.; LaRocque, R.C.; Harris, J.B.; et al. *Vibrio cholerae* serogroup O139: isolation from cholera patients and asymptomatic household family members in Bangladesh between 2013 and 2014. *PLoS Negl. Trop. Dis.* **2015**, *11*, e0004183.
19. Taviani, E.; Grim, C.J.; Chun, J.; Huq, A.; Colwell, R. Genomic analysis of a novel integrative conjugative element in *Vibrio cholerae*. *FEBS Lett.* **2009**, *22*, 3630–3636.

20. de Oliveira Veras, A.A.; da Silva, M.L.; Gomes, J.C.M.; Dias, L.M.; de Sá, P.C.G.; Alves, J.T.C.; Castro, W.; Miranda, F.; Kazuo, E.; Marinho, D.; et al. Draft genome sequences of *Vibrio fluvialis* strains 560 and 539, isolated from environmental samples. *Genome Announc.* **2015**, *1*, e01344-14.
21. Li, Y.; Li, Y.; Fernandez Crespo, R.; Leanse, L.G.; Langford, P.R.; Bossé, J.T. Characterization of the *Actinobacillus pleuropneumoniae* SXT-related integrative and conjugative element ICE Apl2 and analysis of the encoded FloR protein: Hydrophobic residues in transmembrane domains contribute dynamically to florfenicol and chloramphenicol efflux. *J. Antimicrob. Chemother.* **2017**, *1*, 57–65.
22. Okada, K.; Natakuathung, W.; Na-Ubol, M.; Roobthaisong, A.; Wongboot, W.; Maruyama, F.; Nakagawa, I.; Chantaroj, S.; Hamada, S. Characterization of 3 Megabase-Sized Circular Replicons from *Vibrio cholerae*. *Emerg. Infect. Dis*. **2015**, *7*, 1262–1263, doi.org/10.3201/eid2107.141055.
23. Poulin-Laprade, D.; Matteau, D.; Jacques, P.-E.; Rodrigue, S.; Burrus, V. Transfer activation of SXT/R391 integrative and conjugative elements: Unraveling the SetCD regulon. *Nucleic Acids Res*. **2015**, *4*, 2045–2056.
24. López-Pérez, M.; Ramon-Marco, N.; Rodriguez-Valera, F. Networking in microbes: Conjugative elements and plasmids in the genus Alteromonas. *BMC Genomics*, **2017**, *1*, 36.
25. Frey, K.; Bishop-Lilly, K.; Daligault, H.; Davenport, K.; Bruce, D.; Chain, P.; Coyne, S.; Chertkov, O.; Freitas, T.; Jaissle, J.; et al. Full-genome assembly of reference strain *Providencia stuartii* ATCC 33672. *Genome Announ*c. **2014**, *5*, e01082–14.
26. Kuleshov, K.V.; Kostikova, A.; Pisarenko, S.V.; Kovalev, D.A.; Tikhonov, S.N.; Savelieva, I.V.; Saveliev, V.N.; Vasilieva, O.V.; Zinich, L.S.; Pidchenko, N.N.; et al. Comparative genomic analysis of two isolates of *Vibrio cholerae* O1 Ogawa El Tor isolated during outbreak in Mariupol in 2011. *Infect. Genet. Evol.* **2016**, *44*, 471–478.
27. Marin, M.A.; Fonseca, E.L.; Andrade, B.N.; Cabral, A.C.; Vicente, A.C.P. Worldwide occurrence of integrative conjugative element encoding multidrug resistance determinants in epidemic *Vibrio cholerae* O1. *PloS ONE*, **2014**, *9*, e108728.
28. Li, X.Y.; Du, Y.; Du, P.C.; Dai, H.; Fang, Y.J.; Li, Z.P.; Lv, N.; Zhu, B.L.; Kan, B.; Wang, D.C. SXT/R391 integrative and conjugative elements in Proteus species reveal abundant genetic diversity and multidrug resistance. *Sci. Rep.* **2016**, *6*, doi.org/10.1038/srep37372.
29. Bie, L.Y.; Wu, H.; Wang, X.H.; Wang, M.Y.; Xu, H. Identification and characterization of new members of the SXT/R391 family of integrative and conjugative elements (ICEs) in *Proteus mirabilis*. *Int. J. Antimicrob. Agents*, **2017**, *2*, 242–246, doi.org/10.1016/j.ijantimicag.2017.01.045.
30. Fang, Y.; Wang, Y.; Li, Z.; Liu, Z.; Li, X.; Diao, B.; Kan, B.; Wang, D. Distribution and genetic characteristics of SXT/R391 integrative conjugative elements in *Shewanella* spp. from China. *Front. Microbiol.* **2018**, *9*, 920.
31. Siebor, E.; de Curraize, C.; Neuwirth, C. Genomic context of resistance genes within a French clinical MDR Proteus mirabilis: identification of the novel genomic resistance island GI Pmi 1. *J. Antimicrob. Chemother.* **2018**, *7*, 1808–1811.
32. Sugimoto, Y.; Maruyama, F.; Suzuki, S. Draft genome sequence of a *Shewanella halifaxensis* strain isolated from the intestine of Marine Red Seabream (*Pagrus major*), which includes an integrative conjugative element with macrolide resistance genes. *Genome Announc.* **2018**, *16*, e00297-18.
33. Xu, J.; Jia, H.; Cui, G.; Tong, H.; Wei, J.; Shao, D.; Liu, K.; Qiu, Y.; Li, B.; Ma, Z. ICEAplChn1, a novel SXT/R391 integrative conjugative element (ICE), carrying multiple antibiotic resistance genes in *Actinobacillus pleuropneumoniae*. *Vet. Microbiol.* **2018**, *220*, 18–23.
34. Lei, C.-W.; Chen, Y.-P.; Kang, Z.-Z.; Kong, L.-H.; Wang, H.-N. Characterization of a novel SXT/R391 Integrative and Conjugative Element carrying *cfr*, $bla_{CTX-M-65}$, *fosA3* and *aac(6′)-Ib-cr* in *Proteus mirabilis*. *Antimicrob Agents Chemoth.* **2018**, 9,00849–18.
35. Bioteau, A.; Durand, R.; Burrus, V. Redefinition and unification of the SXT/R391 family of integrative and conjugative elements. *Appl. Environ. Microbiol.* **2018,** *13*. 00485–18.
36. Di Noto, G.P.; Jara, E.; Iriarte, A.; Centrón, D.; Quiroga, C. Genome analysis of a clinical isolate of *Shewanella* sp. uncovered an active hybrid integrative and conjugative element carrying an integron platform inserted in a novel genomic locus. *Microbiol.* **2016**, *8*, 1335–1345.
37. Villeneuve, C.; Martineau, C.; Mauffrey, F.; Villemur, R. Complete genome sequences of *Methylophaga* sp. strain JAM1 and *Methylophaga* sp. strain JAM7. *J. Bacteriol.* **2012**, 4126–7.
38. Xu, M.; Fang, Y.; Liu, J.; Chen, X.; Sun, G.; Guo, J.; Hua, Z.; Tu, Q.; Wu, L.; Zhou, J.; et al. Draft genome sequence of *Shewanella decolorationis* S12, a dye-degrading bacterium isolated from a wastewater treatment plant. *Genome Announc.*, **2013**, *6*, e00993–13.

39. Deng, Y.; Chen, C.; Zhao, Z.; Zhao, J.; Jacq, A.; Huang, X.; Yang, Y. The RNA chaperone Hfq is involved in colony morphology, nutrient utilization and oxidative and envelope stress response in *Vibrio alginolyticus*. *PloS ONE*, **2016**, *9*, e0163689.
40. Azarian, T.; Ali, A.; Johnson, J.A.; Mohr, D.; Prosperi, M.; Veras, N.M.; Jubair, M.; Strickland, S.L.; Rashid, M.H.; Alam, M.T.; et al. Phylodynamic analysis of clinical and environmental *Vibrio cholerae* isolates from Haiti reveals diversification driven by positive selection. *MBio*, **2014**, *6*, e01824–14.
41. Bishop-Lilly, K.A.; Johnson, S.L.; Verratti, K.; Luu, T.; Khiani, A.; Awosika, J.; Mokashi, V.P.; Chain, P.S.; Sozhamannan, S. Genome sequencing of 15 clinical Vibrio isolates, including 13 non-O1/non-O139 serogroup strains. *Genome Announc.* **2014**, *5*, e00893–14.
42. Orata, F.D.; Kirchberger, P.C.; Méheust, R.; Barlow, E.J.; Tarr, C.L.; Boucher, Y. The dynamics of genetic interactions between *Vibrio metoecus* and *Vibrio cholerae*, two close relatives co-occurring in the environment. *Genome Biol. Evol.* **2015**, *10*, 2941–2954.
43. Zhu, C.; Sun, B.; Liu, T.; Zheng, H.; Gu, W.; He, W.; Sun, F.; Wang, Y.; Yang, M.; Bei, W.; et al. Genomic and transcriptomic analyses reveal distinct biological functions for cold shock proteins (Vpa CspA and Vpa CspD) in *Vibrio parahaemolyticus* CHN25 during low-temperature survival. *BMC genomics*, **2017**, *1*, 436.
44. Cui, Y.; Yang, X.; Didelot, X.; Guo, C.; Li, D.; Yan, Y.; Zhang, Y.; Yuan, Y.; Yang, H.; Wang, J.; et al. Epidemic clones, oceanic gene pools, and eco-LD in the free living marine pathogen *Vibrio parahaemolyticus*. *Mol. Biol. Evol.* **2015**, *6*, 1396–1410.
45. Kalburge, S.; Polson, S.; Crotty, K.B.; Katz, L.; Turnsek, M.; Tarr, C.; Martinez-Urtaza, J.; Boyd, E. Complete genome sequence of *Vibrio parahaemolyticus* environmental strain UCM-V493. *Genome Announc.* **2014**, *2*, e00159–14.
46. Danin-Poleg, Y.; Raz, N.; Roig, F.J.; Amaro, C.; Kashi, Y. Draft genome sequence of environmental bacterium *Vibrio vulnificus* CladeA-yb158. *Genome Announc.* **2015**, *4*, e00754–15.
47. Roig, F.J.; González-Candelas, F.; Sanjuán, E.; Fouz, B.; Feil, E.J.; Llorens, C.; Baker-Austin, C.; Oliver, J.D.; Danin-Poleg, Y.; Gibas, C.J.; et al. Phylogeny of Vibrio vulnificus from the analysis of the core-genome: Implications for intra-species taxonomy. *Front. Microbiol.* **2018**, *8*, 2613.
48. Tiruvayipati, S.; Bhassu, S.; Kumar, N.; Baddam, R.; Shaik, S.; Gurindapalli, A.K.; Thong, K.L.; Ahmed, N. Genome anatomy of the gastrointestinal pathogen, *Vibrio parahaemolyticus* of crustacean origin. *Gut Pathog.* **2013**, *1*, 37.
49. Yang, Y.-T.; Chen, I.-T.; Lee, C.-T.; Chen, C.-Y.; Lin, S.-S.; Hor, L.-I.; Tseng, T.-C.; Huang, Y.-T.; Sritunyalucksana, K.; Thitamadee, S.; et al. Draft genome sequences of four strains of *Vibrio parahaemolyticus*, three of which cause early mortality syndrome/acute hepatopancreatic necrosis disease in shrimp in China and Thailand. *Genome Announc.* **2014**, *5*, e00816–14.
50. Kumar, R.; Chang, C.-C.; Ng, T.H.; Ding, J.-Y.; Tseng, T.-C.; Lo, C.-F.; Wang, H.-C. Draft genome sequence of *Vibrio parahaemolyticus* strain M1-1, which causes acute hepatopancreatic necrosis disease in shrimp in Vietnam. *Genome Announc.* **2018**, *3*, e01468–17.
51. Diorio, C.; Cai, J.; Marmor, J.; Shinder, R.; DuBow, M.S. An *Escherichia coli* chromosomal ars operon homolog is functional in arsenic detoxification and is conserved in gram-negative bacteria. *J. Bacteriol.* **1995**, *8*, 2050–2056.
52. Ji, G.; Silver, S. Reduction of arsenate to arsenite by the ArsC protein of the arsenic resistance operon of *Staphylococcus aureus* plasmid pI258. *Proc. Natl. Acad. Sci. U.S.A.* **1992**, *20*, 9474–9478.
53. Rosenstein, R.; Peschel, A.; Wieland, B.; Götz, F. Expression and regulation of the antimonite, arsenite, and arsenate resistance operon of *Staphylococcus xylosus* plasmid pSX267. *J. Bacteriol.* **1992**, *11*, 3676–3683.
54. Hedges, R.; Baumberg, S. Resistance to arsenic compounds conferred by a plasmid transmissible between strains of *Escherichia coli. J. Bacteriol.* **1973**, *1*, 459.
55. Bruhn, D.F.; Li, J.; Silver, S.; Roberta, F.; Rosen, B.P. The arsenical resistance operon of IncN plasmid R46. *FEMS Microbiol. Lett.* **1996**, *2–3*, 149–153.
56. Silver, S.; Phung, L.T. Bacterial heavy metal resistance: New surprises. *Annu. Rev. Microb.* **1996**, *1*, 753–789.
57. Tuffin, I.M.; de Groot, P.; Deane, S.M.; Rawlings, D.E. An unusual Tn*21*-like transposon containing an ars operon is present in highly arsenic-resistant strains of the biomining bacterium *Acidithiobacillus caldus*. *Microbiol.* **2005**, *9*, 3027–3039.
58. Davies, M.R.; Shera, J.; Van Domselaar, G.H.; Sriprakash, K.S.; McMillan, D.J. A novel integrative conjugative element mediates genetic transfer from group G streptococcus to other β-hemolytic streptococci. *J. Bacteriol.* **2009**, *7*, 2257–2265.

59. Chen, J.; Madegowda, M.; Bhattacharjee, H.; Rosen, B.P. ArsP: A methylarsenite efflux permease. *Mol. Microbiol.* **2015**, *4*, 625–635.
60. Xia, X.; Postis, V.L.; Rahman, M.; Wright, G.S.; Roach, P.C.; Deacon, S.E.; Ingram, J.C.; Henderson, P.J.; Findlay, J.B.; Phillips, S.E.; et al. Investigation of the structure and function of a *Shewanella oneidensis* arsenical-resistance family transporter. *Mol. Membr. Biol.* **2008**, *8*, 691–701.
61. Xu, C.; Shi, W.; Rosen, B.P. The chromosomal arsR gene of *Escherichia coli* encodes a trans-acting metalloregulatory protein. *J. Biol. Chem.* **1996**, *5*, 2427–2432.
62. Chen, J.; Yoshinaga, M.; Garbinski, L.D.; Rosen, B.P. Synergistic interaction of glyceraldehydes-3-phosphate dehydrogenase and ArsJ, a novel organoarsenical efflux permease, confers arsenate resistance. *Mol. Microbiol.* **2016**, *6*, 945–953.
